# Retroversion of the hemipelvis rather than hypoplastic posterior wall decreases acetabular anteversion in hips affected by Perthes disease

**DOI:** 10.1038/s41598-021-95806-w

**Published:** 2021-08-13

**Authors:** Shijie Liao, Manjun Zhao, Tiantian Wang, Boxiang Li, Chengsen Lin, Anil KC, Zhengtang Liu, Qian Huang, Jinmin Zhao, Rongbin Lu, Xiaofei Ding

**Affiliations:** 1 Department of Orthopedics, the First Affiliated Hospital of Guangxi Medical University, 6 Shuangyong Road, Nanning, Guangxi China; 2 Research Centre for Regenerative Medicine, Guangxi Key Laboratory of Regenerative Medicine, Guangxi Medical University, 22 Shuangyong Road, Nanning, Guangxi China

**Keywords:** Bone, Diseases

## Abstract

The acetabular retroversion has a moderate incidence of 31–60% in all patients of the Perthes disease. It might be caused by posterior wall dysplasia based on recent animal researches. However, some studies support that hemipelvic retroversion is the main factor for the acetabular retroversion. The primary pathological factor of increasing retroversion angle is still controversial anatomically. This study aimed to identify whether there is acetabular retroversion in children with Perthes disease,and to find a method to distinguish version types. Forty children with unilateral Perthes disease who were admitted to our hospital from January 1, 2012 to December 31, 2018 were enrolled, and 40 controls were matched based on sex and age. The acetabular anteversion angle (AAA), internal wall anteversion angle (IWAA), anterior wall height of the acetabulum (A), acetabular posterior wall height (P), and acetabular width (W) were assessed on computed tomography (CT) at the level of the femoral head center. The acetabular wall difference index (AWDI; AWDI = P-A)/W*100) was calculated. The mean AAA was significantly lower in Perthes disease hips (10.59 (8.05–12.46)) than in contralateral hips (12.04 (9.02–13.33)) (p = 0.002) but did not differ from control hips (9.68 ± 3.76) (p = 0.465). The mean IWAA was significantly lower in Perthes hips (9.16 ± 3.89) than in contralateral hips (11.31 ± 4.04) (p = 0.000) but did not differ from control hips (9.43 ± 3.82) (p = 0.753). The mean AWDI did not differ between Perthes hips (0.41 ± 4.94) and contralateral hips (− 1.12 (− 4.50, 2.17)) (p = 0.06) or control hips (− 0.49 ± 5.46) (p = 0.437). The mean W was significantly higher in Perthes hips (44.61 ± 5.06) than in contralateral hips (43.36 ± 4.38) (p = 0.000) but did not differ from control hips (45.02 ± 5.01) (p = 0.719). The mean A and P did not differ between Perthes hips and contralateral hips or control hips. Correlation analysis of all hip joints revealed a significant correlation between AAAs and IWAAs (r = 0.772; r = 0.643; r = 0.608; and r = 0.540). Linear regression analysis revealed that AAAs increased with IWAAs. Multiple linear regression showed that IWAAs and AWDIs have good predictive value for AAAs in both Perthes and control hips (R^2^ = 0.842, R^2^ = 0.869). In patients with unilateral Perthes disease, the affected acetabulum is more retroverted than that on the contralateral side, which may be caused by hemipelvic retroversion. The measurements in this study could distinguish the form of acetabular retroversion. IWAAs and AWDIs can be used as new observations in future studies of acetabular version.

## Introduction

Perthes disease is caused by blood supply dysfunction in the femoral head in childhood. Currently, the main lesion is believed to be located in the femoral head, but some studies have shown that Perthes disease can also cause morphological changes in the acetabulum^1–4^. A gradual decrease in acetabular anteversion angle may led to acetabular retroversion. Ezoe et al. investigated the incidence of acetabular retroversion in different hip diseases; the authors reported that 42% of patients with Perthes disease had acetabular retroversion, and it was associated with poor disease prognosis^5^. Subsequent studies have shown that 31–60% of Perthes patients developed acetabular retroversion^6–8^. Reynolds et al. first proposed that acetabular retroversion was the main cause of hip pain in 1999^9^, and it was also reported to be an important cause of pincer femoroacetabular impingement, labrum injury and early osteoarthritis^10–15^.

In 2014, Shapiro et al.^16^ studied the pathological changes of the acetabular side of Perthes disease in animal models and noted that after 8 weeks of intracapsular circumferential ligation of the femoral neck in young pigs, the articular cartilage and adjacent bone of the acetabulum on the surgical side underwent secondary morphological changes after femoral head necrosis. In 2017, Upasani et al.^17^ repeated the same animal model study. They found that specific changes took place in the morphology of the operative acetabulum 8 weeks after surgery and observed a significant reduction in the coverage angle of the upper, posterior and lower quadrants of the acetabulum, as well as a decrease in the acetabular anteversion angle (AAA) and tilt angle, showing acetabular retroversion. The changes were similar to those seen in the acetabulum in Perthes disease. The authors hypothesized that the necrosis of the femoral head may affect the development of the posterior wall of the acetabulum during puberty and that acetabular retroversion may occur when the posterior coverage angle is reduced.

However, the abnormal development of the anterior and posterior walls of the acetabulum is not the only cause of acetabular retroversion, hemipelvic retroversion can also cause acetabular retroversion^18–20^. Hemipelvic retroversion may appear as the positive prominence of the ischial spine sign (PRIS). Larson et al.^21^ researched acetabular retroversion and PRIS in children with Perthes disease and found skeletally immature hips in 90% (37/41) of children with a positive PRIS sign but in only 32% (16/50) of children in the normal control group. Additionally, the positive PRIS sign rate was 90% (9/10) in the early stage of Perthes disease, which showed that acetabular retroversion was probably caused by hemipelvic retroversion in Perthes disease patients.

Presently, it is unclear whether acetabular retroversion in Perthes patients is caused by the dysplasia of the posterior acetabular wall or by hemipelvic retroversion, and there is no measurement method to distinguish the changes in the anatomical pathology. Although radiologic parameters such as the crossover sign, posterior wall sign and PRIS are currently the most commonly used to determine acetabular reversion, these indicators are affected by posture and photograph conditions, as well as by observer experience. The posterior wall sign and crossover sign are not applicable to children whose acetabular walls are not fully ossified. These parameters are not suitable for the quantitative analysis of the degree of retroversion and have little effect on guiding treatment. Although the CT or magnetic resonance imaging (MRI) cross-sectional measurement of the AAA can be used to quantitatively analyze the extent of acetabular retroversion, it cannot be used to distinguish the pathological anatomic form of acetabular retroversion.

Therefore, our study aimed to determine if there is acetabular retroversion and to find a measurement method to explore which anatomical deformity causes decreased acetabular anteversion that finally leads to acetabular retroversion in children with Perthes disease.

## Materials and methods

### Materials

This study retrospectively analyzed children with Perthes disease who were admitted to our hospital (outpatient and inpatient) from January 1, 2012, to December 31, 2018. According to the inclusion and exclusion criteria, the imaging data of the children were collected from the imaging system of our hospital as the case group.

The inclusion criteria were as follows: (1) the patient had CT images of the bony pelvis without surgery (preoperative and conservative treatment) and X-rays of the bony pelvis no more than three months before or after CT examinations; (2) the patient suffered in only a unilateral hip; and (3) the CT thickness of the cross section of the patient was less than 3.0 mm^22^.

The exclusion criteria included the following: (1) the pelvic inclination measured on the positioning film was greater than 3°^23^ (the included angle between the apical line of the acetabulum and the positioning line); (2) the pelvis was excessively tilted forward or backward during the CT scan (the distance from the sacrococcygeal joint to the pubic symphysis on the positioning film was greater than 5 cm or less than 1 cm^24^); (3) there were bilateral lesions; (4) misdiagnosed cases; and (5) there were other types of femoral head necrosis caused by trauma, hormones, postoperative developmental dysplasia of the hip (DDH), etc.

### Methods

#### The case group

**Figure 1:**
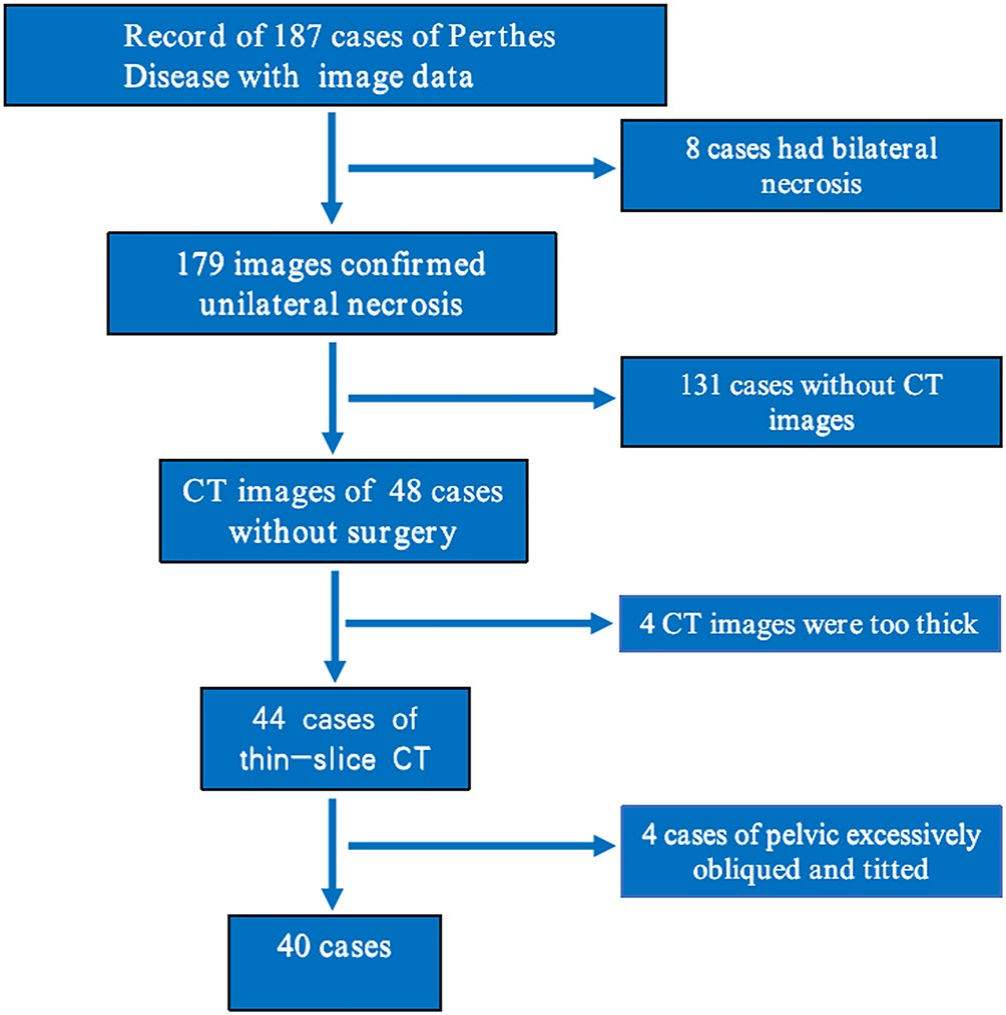
screening flow chart of case group. (Adobe Illustrator Cs6, Version 16.0.0, URL: https://www.adobe.com/cn/products/illustrator.html).

Records from 187 Perthes disease patients with imaging data were found in the outpatient and inpatient systems of our hospital. After consulting the picture archiving and communication system (PACS) imaging system of our hospital, 8 patients had bilateral necrosis. The remaining 179 patients were confirmed to have unilateral Perthes disease by imaging data. Next, we consulted the CT data excluded 131 patients without CT images. Among the remaining 48 patients, 4 patients were excluded based on having CT images with thicknesses greater than 3 mm, and 4 patients were excluded due to excessive pelvic inclination and tilting. Finally, 40 children with unilateral Perthes disease were included in the case group (Fig. 1).

#### The control group

According to the final number of included cases, 40 children were matched at a 1:1 ratio according to age, sex and examination time through the PACS system of our hospital and were included in the control group. The inclusion criteria of the control group were as follows: (1) the control group included children who underwent abdominal CT at our hospital; (2) CT included the bilateral hip joint and proximal femur; (3) the gender was the same as that of the case group, the age difference was less than 0.5 years, and the examination date difference was less than 6 months; and (4) the CT thickness of the cross section was less than 3 mm. The exclusion criteria were as follows: (1) malignant tumors; (2) premature epiphyseal closure; (3) excessive pelvic inclination and excessive pelvic tilting (same criteria as above); (4) hip lesions; and (5) neuromuscular disease.

#### Measuring tools

AnnetPACS Version 2.0.0.1, PicPick (All-in-one Design Tool Version 4.2.1,2016 NGWIN), Microsoft Windows 6.1.

#### Measuring methods

**Figure 2:**
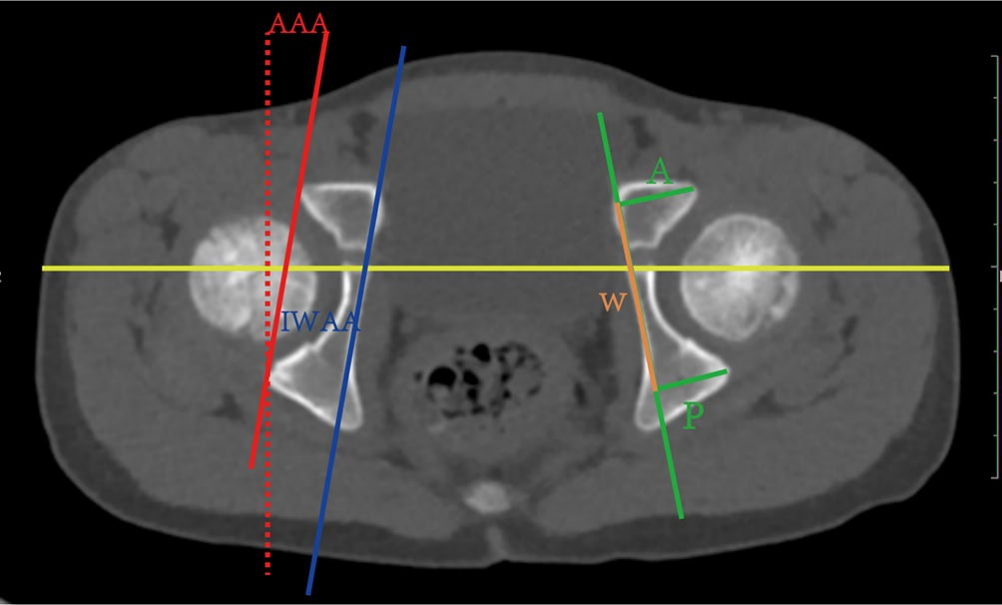
Measurement methods: The baseline (yellow line) was obtained by connecting the center of the bilateral Y-shaped cartilage, and the vertical baseline was used as the reference line (red dotted line). The anterior and posterior margins of the bone acetabulum were connected (red solid line), and the angle of intersection with the reference line was the AAA. A parallel line (blue line) was made through the straight bone cortex of the inner wall of the pelvis, and the angle of intersection with the reference line was the IWAA. The distance from the highest point of the anterior and posterior margins of the bony acetabulum to the straight line of the pelvic wall was the A (green line) and P (green line), respectively. The distance from the point of the projection of the highest point of the anterior and posterior walls onto the straight line of the pelvic wall was the W (brown line).

We found that a straight line could be drawn along the inner wall of the pelvis on the transverse CT scan through the central plane of the bilateral femoral head, which could indicate the horizontal orientation of the hemipelvis. Therefore, cross-sectional CT through the maximal plane of the bilateral femoral head was selected (there was a good correlation between the middle and top acetabular anteversion^25^); the adjusting window width was 700, and the window level was 3200. Measurement of the AAA: The baseline was obtained by connecting the center of the bilateral Y-shaped cartilage^22^, and the perpendicular line was the reference line. The anterior and posterior margins of the bone acetabulum were connected, and the angle of intersection with the reference line was the AAA. Internal wall anteversion angle (IWAA): A straight line was made through the lateral wall of the pelvis at the level of the bone (1/3 posterior to the pubis and 1/2 anterior to the ischium), and the angle at which the line intersected the reference line was the IWAA. Anterior and posterior wall height of the pelvis (A and P, respectively): The distance from the highest point of the anterior and posterior margins of the bone acetabulum to the straight line of the bone cortex of the inner wall of the pelvis was measured as the height of the A and the P, respectively. Acetabular width (W): The distance between the highest point of the anterior wall and the highest point of the posterior wall was the acetabular width. Formula for calculating the difference index of the acetabular wall: (AWDI) = (P-A)/W*100. The bilateral acetabulum was measured and recorded in both the case and control groups. In the case group, the affected side and the contralateral side were recorded; in the control group, the control side and the uninvolved side were recorded (Fig. 2).

To evaluate the reliability of the measurement, 5 children were randomly selected from the case group and the control group, and the bilateral acetabulum was measured at different times by the same observer (Zhao). In addition, the bilateral acetabulum of the 10 children was also measured by another observer (Ding). Thus, the reliability of the measured data was tested.

### Statistical analysis

Statistical analysis of the data was performed by SPSS 20.0 software and Graph Pad Prism7.00. The intragroup correlation coefficient (ICC) was used for data reliability analysis. One-way random and ICC (1,1) were used to examine the retest reliability. Bidirectional randomness, absolute consistency, and ICC (2,1) were used to evaluate interobserver reliability. The Shapiro–Wilk test was used to test the normality of the data. Measurement data: Normal data are described as the mean ± the standard deviation (SD), while non-normal data are described as the median (p25, p75). An independent sample T test was used to compare normal data sets. Intragroup comparisons were performed using a paired sample T test. The Mann–Whitney U test was used to compare non-normal data sets. The Wilcoxon rank sum test was used for intragroup comparisons. Spearman correlation was used for rank data and single factor correlation analysis, while Pearson correlation was used for numerical data. Linear regression and multiple linear regression were used to analyze the factors affecting acetabular reversion.

### Ethics approval and consent to participate

We confirmed that all methods were carried out in accordance with relevant guidelines and regulations. We confirm that all experimental protocols were approved by Ethics Committee of the First Affiliated Hospital of Guangxi Medical University. The informed consent was obtained from parents of all participants.

## Results

**Figure 3:**
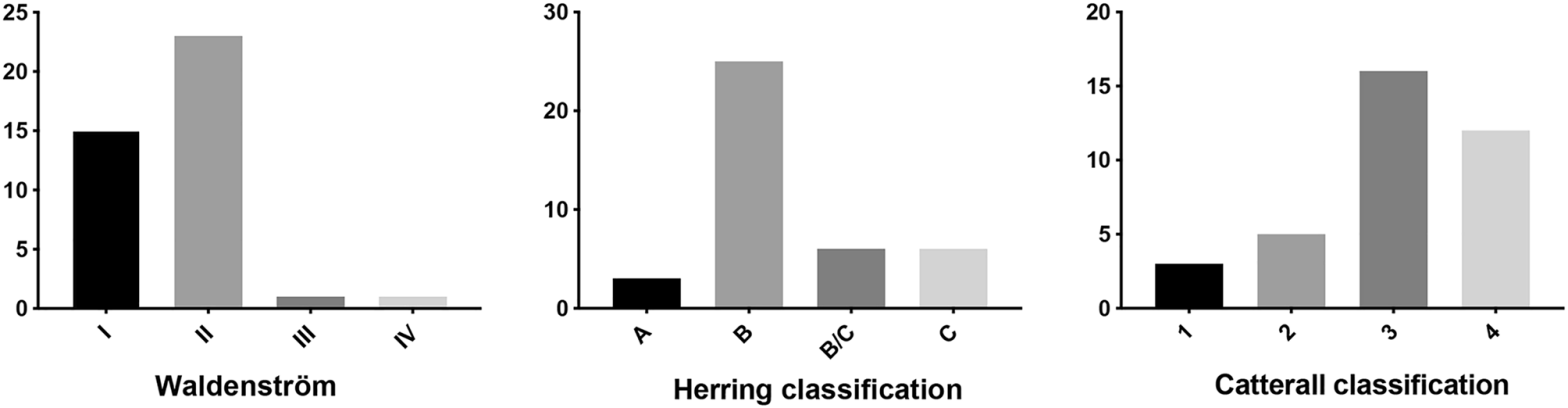
Waldenström, Herring classification and Catterall classification in case group.

After the exclusion criteria were applied, 40 children (33 boys and 7 girls) were included in the disease group. None of the 40 children had Y-shaped cartilage closure. The boys had a median age of 8.27 ± 2.38 years, and the median age the girls was 8.34 ± 2.62 years at the time of CT testing. There were 15 patients with a left affected hip and 25 patients with a right affected hip in the case group. During CT examinations, there were 15 patients with Waldenström stage I, 23 with stage II, and 2 with stage III. For the modified lateral pillar classification, there were 3 people in group A, 25 people in group B, 6 people in group B/C, and 6 people in group C. For the Catterall classification, there were 3 people in group I, 5 people in group II, 1 person in group III, and 12 people in group IV (the Catterall classification could not be determined for 4 children without frog-type radiographs) (Fig. 3).

Similarly, the control group included 40 children (33 boys and 7 girls), the median age of the boys at the time of CT was 8.33 ± 2.36 years, and the median age of the girls was 8.26 ± 2.43 years. The age difference between the control group and the case group was 0.03 ± 0.29 years, and the difference in the examination time was 0.42 ± 2.24 months. Similar to the disease group, none of the 40 children had Y-shaped cartilage closure, and 57.5% (23/40) of the control group had urinary tract disease. There were 12 patients with pelvic ureteral stenosis, 2 patients with hydronephrosis, 1 patient with a renal cyst, 6 patients with urinary calculi, 1 patient with recurrent kidney, 1 patient with urethral trauma, 5 patients with abdominal pain, 1 patient with abdominal trauma, and 2 patients with ovarian cyst pancreatitis. There was 1 patient with congenital megacolon, 1 patient with intestinal torsion, 1 patient with teratoma, 1 patient with lipoma, 1 patient with enteritis, 1 patient with duodenitis, and 2 patients with lymphadenopathy.

To test the reliability of the observed indicators, the measurements of ten children (20 hips) who were randomly selected by two observers were used to calculate the test–retest reliability and interobserver reliability (Table 1). The same observer's measurement data from two times had excellent test–retest reliability (0.90 < ICC). Observers had excellent reliability in the A, P, and W (0.90 < ICC). They also had good reliability (0.75 < ICC) for the measurements of the IWAA and AAA^26^.

**Table 1:** Reliability of CT acetabular measurements (n = 20).

Measurement	Intraobserver	Interobserver
A	0.936 (0.849–0.974)	0.913 (0.794–0.965)
P	0.940 (0.857–0.976)	0.959 (0.900–0.983)
W	0.984 (0.962–0.994)	0.959 (0.898–0.984)
AAA	0.932 (0.840–0.972)	0.848 (0.657–0.937)
IWAA	0.931 (0.837–0.972)	0.839 (0.625–0.934)

*The values are given as the intraclass correlation coefficient, with the 95% confidence interval in parentheses.

There was no statistically significant difference in any measured parameters when the disease groups were compared with the controls. Not only the affected and the control acetabulum but also the contralateral and uninvolved sides were not significantly different for any measured parameters (Table 2). The AAA on the affected or control acetabular sides of the disease group and the control group was not significantly different (10.59 (8.05–12.46) vs. 9.68 ± 3.76, p = 0.465 and 12.04 (9.02–13.33) 10.43 ± 3.15, p = 0.112). At the same time, there was no significant difference in the IWAA on either side of the disease group and control group (9.16 ± 3.89 vs. 9.43 ± 3.82, p = 0.753 and 11.31 ± 4.04 vs. 10.22 ± 3.55, p = 0.202). There was also no significant difference in the AWDI (0.41 ± 4.94 vs. − 0.49 ± 5.46, p = 0.437 and − 1.12 (− 4.50–2.17) vs. 0.04 ± 6.04, p = 0.795), and there was no significant difference in the A, P and W between the two groups of children.

**Table 2:** Comparison of case group and Control group.

		Case group	Control group		p
**Affected side**					
	A (mm)	18.32 ± 2.60	19.07 (16.36–20.14)	0.501^△^	
	P (mm)	17.63 (16.18–20.18)	18.04 (16.13–19.29)	0.817^△^	
	W(mm)	44.61 ± 5.06	45.02 ± 5.01	0.719^▲^	
	AWDI	0.41 ± 4.94	− 0.49 ± 5.46	0.437^▲^	
	AAA (°)	10.59 (8.05–12.46)	9.68 ± 3.76	0.465^△^	
	IWAA (°)	9.16 ± 3.89	9.43 ± 3.82	0.753^▲^	
**Contralateral side**					
	A (mm)	18.55 ± 2.47	18.41 ± 2.77	0.800^▲^	
	P (mm)	17.81 (16.01–19.62)	17.95 (16.08–19.23)	0.840^△^	
	W (mm)	43.36 ± 4.38	45.12 ± 4.92	0.097^▲^	
	AWDI	− 1.12 (− 4.50–2.17)	0.04 ± 6.04	0.795^△^	
	AAA (°)	12.04 (9.02–13.33)	10.43 ± 3.15	0.112^△^	
	IWAA (°)	11.31 ± 4.04	10.22 ± 3.55	0.202^▲^	

“▲” use independent sample t test. “△” uses Mann Whitney U test. Normal data, described by mean ± SD, non-normal data, described by median (P25-P75).

We then compared the above parameters of the affected acetabulum and the contralateral acetabulum in the disease group. Interestingly, there were statistically significant differences in the acetabular anteversion and the anteversion angle of the inner wall of the pelvis. The AAA of the affected side of the children was smaller than that of the contralateral side (10.59 (8.05–12.46) vs. 12.04 (9.02–13.33), p = 0.002). The IWAA was reduced in the affected side compared with the contralateral side (9.16 ± 3.89 vs. 11.31 ± 4.04, p = 0.000). Although the AWDI on both sides of the case group was not significantly different (0.41 ± 4.94 vs. 1.12 (− 4.50, 2.17), p = 0.060), the measurement showed that the width the acetabulum of the affected side of was larger than that of the contralateral side (44.61 ± 5.06 vs. 43.36 ± 4.38, p = 0.000), and the difference was statistically significant. There was no significant difference between the front wall height and the rear wall height (Table 3).

**Table 3:** Comparison of affected side and contralateral side.

	Affected side	Contralateral side	*p*
**Case group**			
A (mm)	18.32 ± 2.60	18.55 ± 2.47	0.212^▲^
P (mm)	17.63 (16.18–20.18)	17.81 (16.01–19.62)	0.255^△^
W (mm)	44.61 ± 5.06	43.36 ± 4.38	0.000^▲^*
AWDI	0.41 ± 4.94	− 1.12 (− 4.50,2.17)	0.060^△^
AAA (°)	10.59 (8.05–12.46)	12.04 (9.02–13.33)	0.002^△^*
IWAA (°)	9.16 ± 3.89	11.31 ± 4.04	0.000^▲^*
**Control group**			
A (mm)	19.07 (16.36,20.14)	18.41 ± 2.77	0.682^△^
P (mm)	18.04 (16.13–19.29)	17.95 (16.08–19.23)	0.375^△^
W (mm)	45.02 ± 5.01	45.12 ± 4.92	0.622^▲^
AWDI	− 0.49 ± 5.46	0.04 ± 6.04	0.326^▲^
AAA (°)	9.68 ± 3.76	10.42 ± 3.15	0.138^▲^
IWAA (°)	9.43 ± 3.82	10.22 ± 3.55	0.078^▲^

“▲” use paired t test. “△” uses Wilcoxon rank test. “*” indicates statistical significance. Normal data, described by mean ± standard deviation. Non-normal data, described by median (P25, p75).

To further explore the factors influencing the AAA, IWAA, and AWDI, we performed univariate analysis. The results suggested that there was no significant correlation between age, gender, Waldenström stage, Herring lateral column classification, and Catterall classification (Table 4). To analyze the factors influencing the AAA, IWAA, AWDI, A, P, and W were analyzed in the two groups of children (Table 5). A moderately high correlation was found between the AAA and IWAA in both groups (r = 0.772, p = 0.000; r = 0.643, p = 0.000; r = 0.608, p = 0.000; and r = 0.540, P = 0.000). Unfortunately, the AAA was moderately correlated with the AWDI in the control group. The A, P and W were not significantly related to the AAA.

**Table 4:** One-way correlation analysis.

	AAA		IWAA		AWDI	
	r	p	r	p	r	p
Age	0.091	0.576	− 0.053	0.745	0.301	0.059
Gender	0.259	0.106	0.197	0.224	0.043	0.793
Waldenström	− 0.007	0.966	− 0.099	0.543	0.028	0.865
Herring classification	− 0.256	0.111	− 0.092	0.573	− 0.206	0.203
Catterall classification	− 0.174	0.310	− 0.143	0.406	− 0.087	0.612

**Table 5:** Correlation Analysis of AAA.

	Affected side		Contralateral side	
	r	*p*	r	*p*
**Case group**				
AAA-A	0.187	0.249	0.056	0.733
AAA-P	0.224	0.165	0.240	0.136
AAA-W	-0.024	0.882	-0.055	0.738
AAA-AWDI	0.134	0.408	0.215	0.182
AAA-IWAA	0.772	0.000*	0.643	0.000*
**Control group**				
AAA-A	-0.106	0.515	0.064^△^	0.696
AAA-P	0.259	0.107	0.218	0.177
AAA-W	-0.042^△^	0.796	-0.036^△^	0.826
AAA-AWDI	0.405^△^	0.010*	0.246^△^	0.126
AAA-IWAA	0.608^△^	0.000*	0.540^△^	0.000*

“^△^” indicates the use of Person, “*” indicates statistical significance.

**Figure 4:**
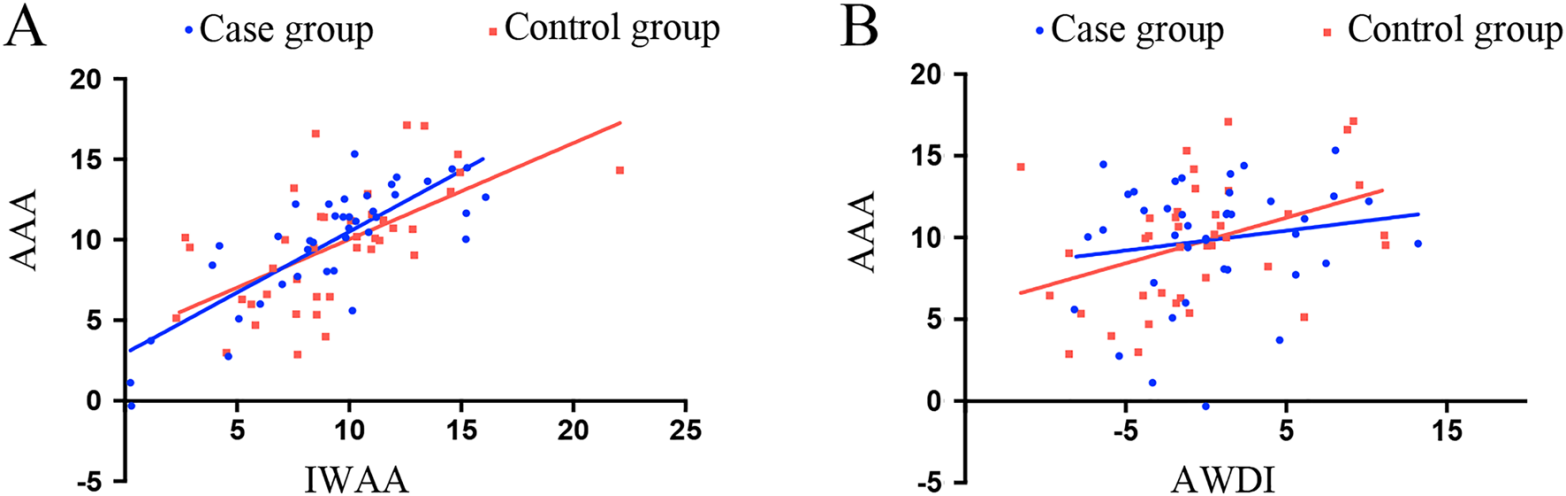
single factor linear regression of IWAA and awdi in patients and controls. Case group: AAA = 0.7563*IWAA + 2.941 R2 = 0.6456 AAA = 0.1215*AWDI + 9.815 R2 = 0.02686. Control group: AAA = 0.5985*IWAA + 4.042 R2 = 0.3703 AAA = 0.2787*AWDI + 9.823 R2 = 0.163. Multiple linear regression equations of two groups of affected sides. Case group: AAA = 0.355*AWDI + 0.904*IWAA + 1.434 R2 = 0.842. Control group: AAA = 0.535*AWDI + 0.907*IWAA + 1.394 R2 = 0.869.

**Figure 5:**
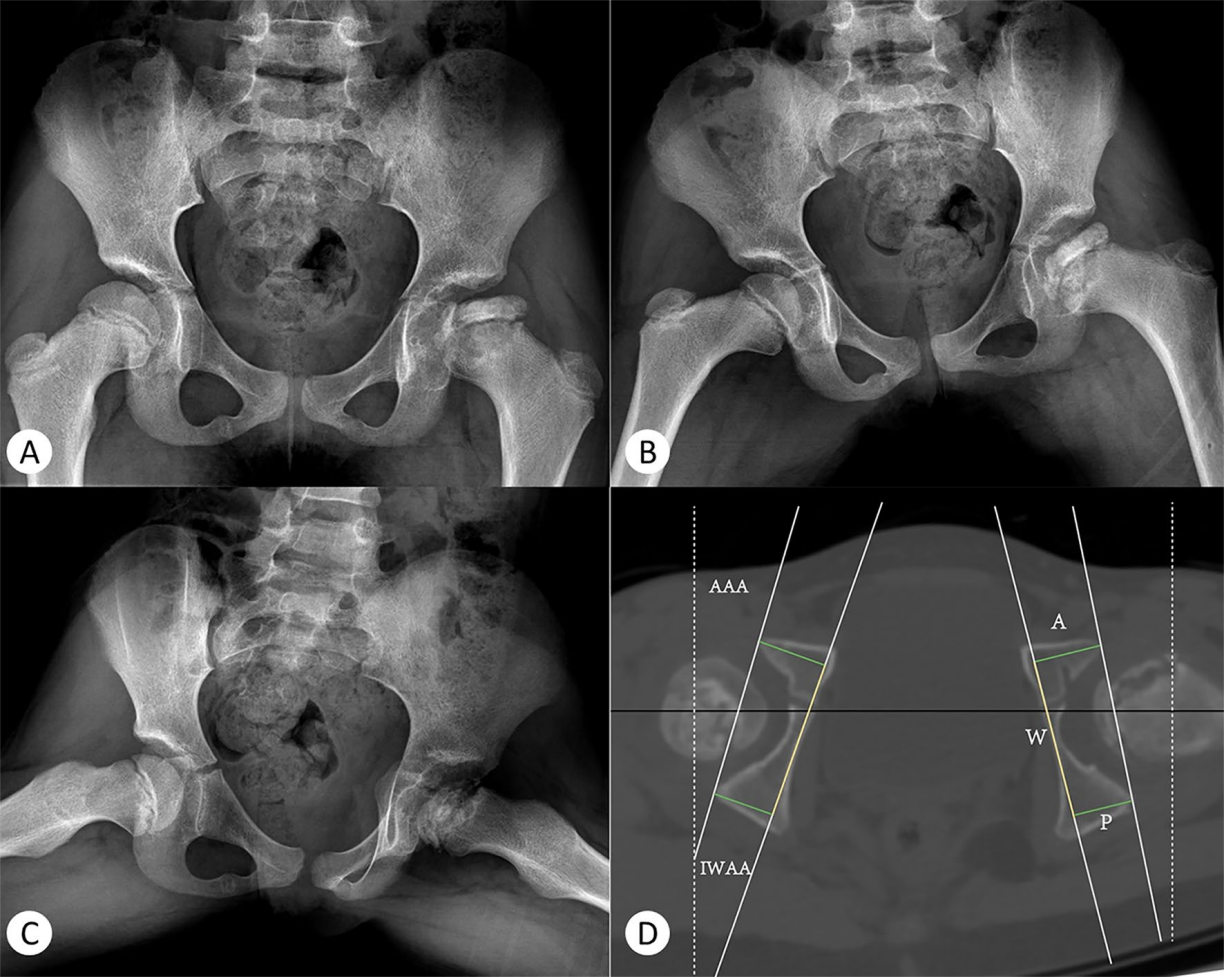
An eight-year-old girl with Legg–Calve–Perthes disease in the left hip. The anteroposterior pelvis radiograph (**A**), Von-Rosen pelvis radiograph (**B**) and frog pelvis radiograph (**C**) show that patient was in Waldenström II stage, Herring B stage and Catterall III stage.

**Figure 6:**
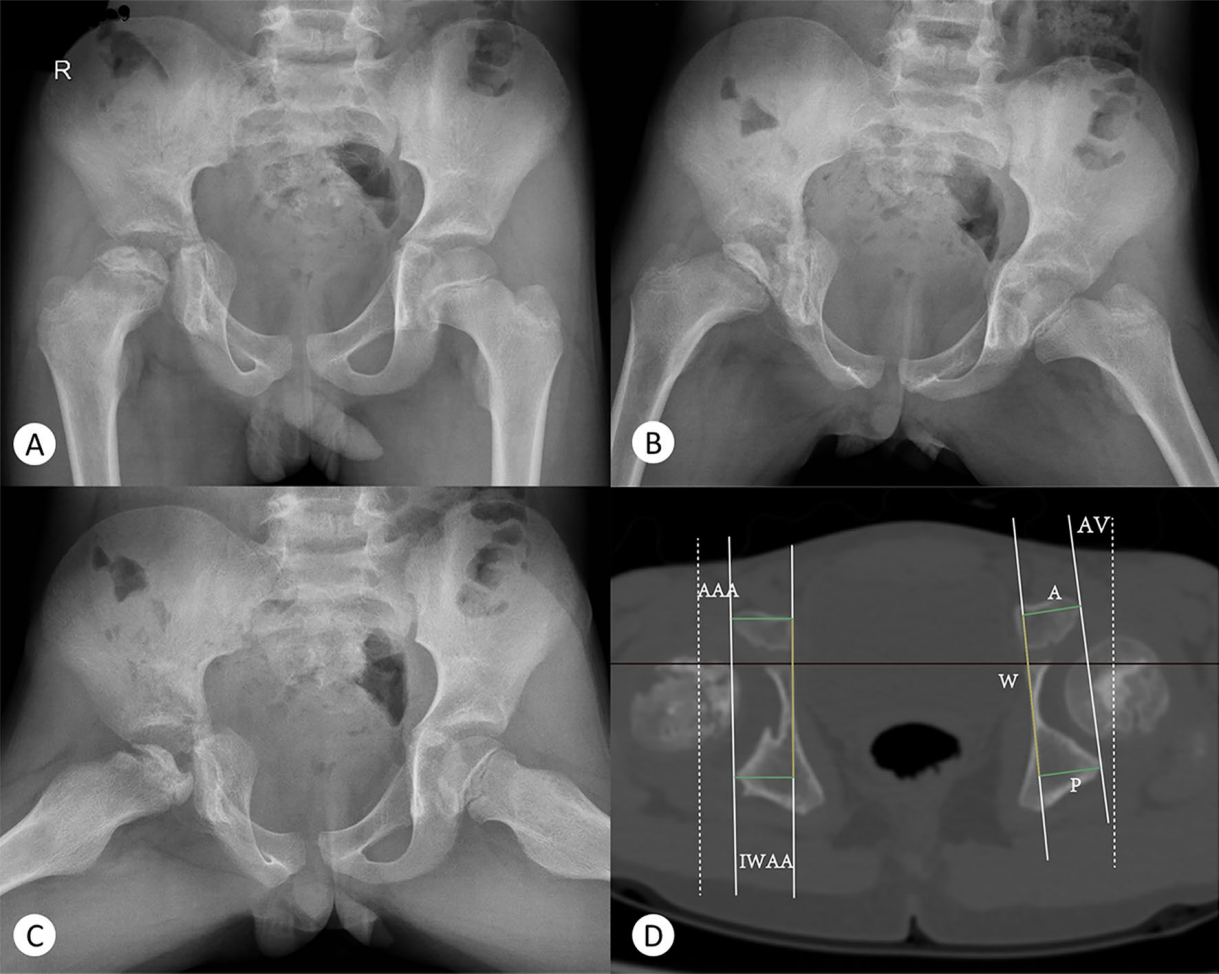
An eight-year-old boy with Legg-Calve-Perthes disease in the right hip. The anteroposterior pelvis radiograph (**A**), Von-Rosen pelvis radiograph (**B**) and frog pelvis radiograph (**C**) show that patient was in Waldenstrom II stage, Herring B stage and Catterall III stage.

Because the IWAA and AWDI had a good correlation with the AAA, next, the IWAA and AWDI of the affected side of the case group and the control group were used to calculate the linear regression equations of the AAA, respectively. For the disease group, AAA = 0.7563 * IWAA + 2.941, R^2^ = 0.6456; and AAA = 0.1215 * AWDI + 9.815, R^2^ = 0.02686. For the control group, AAA = 0.5985 * IWAA + 4.042, R^2^ = 0.3703; and AAA = 0.2787 * AWDI + 9.823, R^2^ = 0.1636. The AAA was more highly correlated with the IWAA than with the AWDI. The multiple linear regression equation of the AAA was calculated using the IWAA and AWDI. For the disease group, AAA = 0.355 * AWDI + 0.904 * IWAA + 1.434, R^2^ = 0.842. For the control group, AAA = 0.535 * AWDI + 0.907 * IWAA + 1.394, R^2^ = 0.869. In both the control and case groups, the simultaneous use of the IWAA and AWDI had good predictive value for the AAA (Fig. 4). Typical cases are shown in Figs. 5 and 6, and their data measurements are shown in Tables 6 and 7.

**Table 6:** Fig. 5-D data measurement.

	**Right**	**Left**
AAA **(°)**	15.67	12.65
IWAA **(°)**	20.65	16.09
A(mm)	20.77	19.80
P(mm)	17.93	17.70
W(mm)	43.43	43.26
AWDI	-6.54	-4.85

**Table 7:** Fig. 6-D data measurement.

	Right	Left
AAA (°)	-0.33	7.14
IWAA (°)	0.29	4.42
A (mm)	16.19	17.80
P (mm)	16.19	17.01
W (mm)	43.60	44.16
AWDI	0	-1.79

## Discussion

Currently, the pathoanatomical location that decreases acetabular anteversion angle or even causes acetabular retroversion in Perthes disease is unclear, and there is no direct measurement method to distinguish the two forms of acetabular retroversion. In this study, the anteversion of the acetabulum, anteversion of the internal wall of the pelvis, AWDI, A, P and W were measured on cross-sectional CT. The anteversion of the internal wall of the pelvis was used as the index to describe the baseline of the acetabulum, and the AWDI was used as the index to describe the development of the acetabulum on the horizontal plane to distinguish the decrease in AAA that causes Perthes disease. Our results confirmed that the AAA of the affected side of the case group was smaller than that of the contralateral side, as was the IWAA, but the AWDI was the same on both sides. Correlation analysis also showed that the AAA can be predicted using both the IWAA and the AWDI. Therefore, the reduction in the anteversion angle of the affected side of the acetabulum might be caused by hemipelvic retroversion.

Acetabular retroversion can be caused by the abnormal development of the anterior and posterior walls of the acetabulum and the retroversion of the hemipelvis^18,19^. However, there is no imaging index that can distinguish the acetabular retroversion caused by the two kinds of pathological anatomy. The crossover sign, posterior wall sign and PRIS are the most commonly used X-ray qualitative indexes to evaluate acetabular retroversion^20^. Later, some scholars also proposed that the acetabular wall index and anterior posterior wall ratio can also reflect acetabular retroversion^27,28^. However, due to being X-ray observations, these indicators are affected by the position, shooting conditions and experience of the observer^29,30^. The crossover sign and posterior wall sign are not suitable for children and adolescents whose acetabular wall is not completely ossified. The PRIS seems to indicate retroversion of the hemipelvis, but it does not distinguish whether the retroversion is associated with acetabulum wall dysplasia and cannot be quantitatively analyzed. The measurement of the AAA on MRI or CT cross-sectional images is a quantitative analysis method to evaluate the degree of acetabulum retroversion^31^.

However, the AAA can only reflect the opening of the acetabulum in the horizontal plane in entirety but cannot distinguish the position causing acetabular retroversion. The acetabular sector angle reflects the inclusion of the anterior and posterior walls of the acetabulum to the femoral head in the horizontal plane, and it is also difficult to distinguish whether acetabular retroversion is caused by hemipelvic retroversion or the abnormal development of the anterior and posterior walls of the acetabulum. Currently, there is no good method to distinguish the anatomic site of acetabular retroversion.

**Figure 7:**
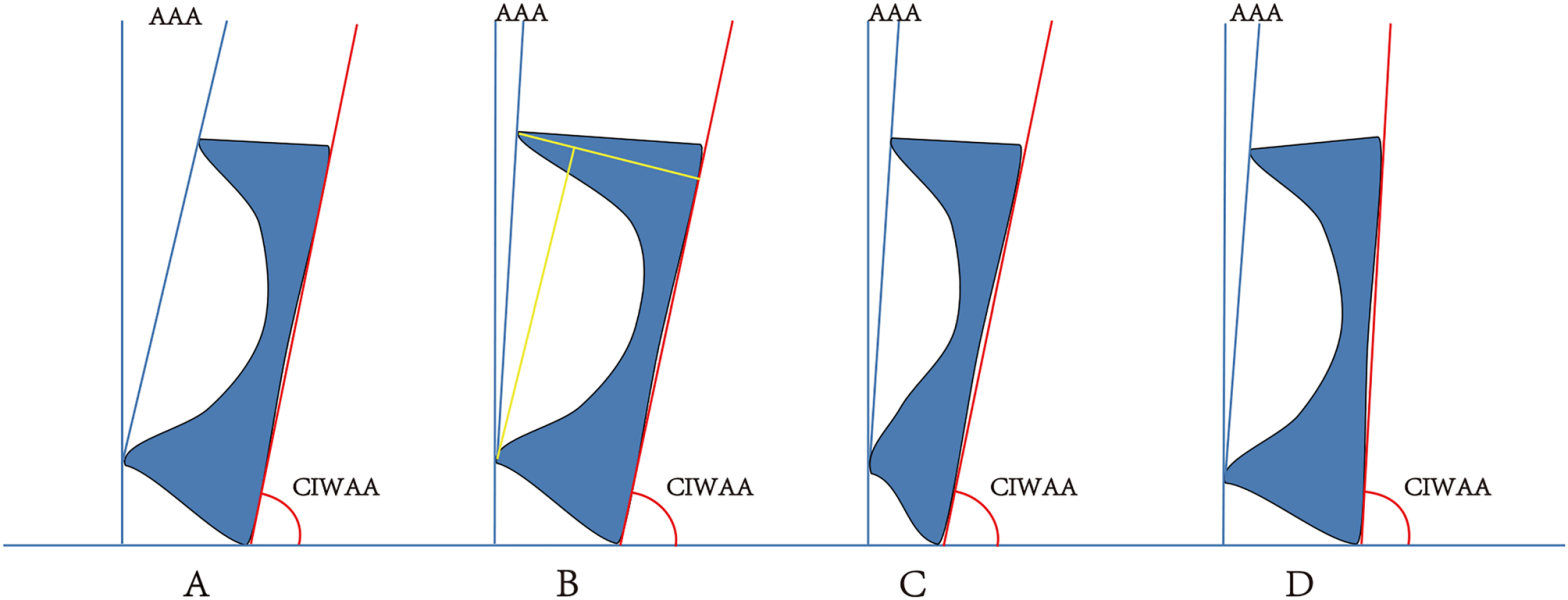
Schematic diagram of different types of acetabulum retroversion. (**A**) Normal acetabulum; (**B**) Protrusion of anterior wall and normal posterior wall; (**C**) Normal anterior wall and dysplasia of posterior wall; (**D**) Normal anterior and posterior wall and retroversion of hemipelvis. AAA is acetabulum anteversion; CIWAA is complement angle of pelvis anteversion (CIWAA = 90-IWAA). (Adobe Illustrator Cs6, Version 16.0.0, URL: https://www.adobe.com/cn/products/illustrator.html).

Acetabular anteversion is affected by the development of the acetabular wall and by the anteversion of the pelvic wall^19^ (Fig. 7). The AAA can only describe the acetabular opening in entirety. In this study, the AWDI was used to evaluate the influence of the anterior and posterior walls of the acetabulum and acetabular width on acetabular AAA opening in the horizontal plane, and the IWAA was used to evaluate hemipelvic rotation in the horizontal plane. The results showed that both measurements had good reliability for one observer and between observers, and simultaneously using the IWAA and the AWDI has good predictive value for the AAA. Similar to this study, Musielak et al.^32^ used the iliac opening angle to indicate the opening direction of the hemipelvis in the horizontal plane, but the morphological analysis of the open angle of the ilium for the acetabulum part of the hemipelvis was insufficient. Fuji et al.^33^ also analyzed the rotation of the ilium on three levels, but his measurement method failed to analyze the influence of the development of the anterior and posterior walls of the acetabulum on the acetabular opening. The analysis of the anteversion of the inner pelvic wall and the AWDI can be used to determine the anatomic site of acetabular retroversion.

In 2008, Kalberer et al.^20^ proposed that acetabular retroversion can also be caused by hemipelvic retroversion and not only by the hypoplasia of the posterior wall or the overdevelopment of the anterior wall. Subsequently, some scholars have shown that a high proportion of patients with Perthes disease have a positive ischial spine sign^8,21^. This suggests that acetabular retroversion in Perthes disease may be caused by hemipelvic retroversion. Musielak et al.^32^ also found that the structure of the hemipelvis is closely related to the direction of the acetabulum, and the acetabulum will also be rotated back in proportion when the hemipelvis is rotated back in the horizontal plane. In this study, the AAA of the affected side of the case group was smaller than that of the contralateral side, and the IWAA of the affected side was smaller than that of the contralateral side. However, there was no difference between the two sides for the AWDI, which also suggests that the decrease in the anteversion of the acetabulum of the affected side may be caused by hemipelvic retroversion.

Not only will the abnormal anatomical structure affect the opening direction of the acetabulum, but the position of the pelvis can also be affected by different positions and shooting conditions, which leads to changes in the opening direction of the acetabulum. Van Bosse et al.^34^ found that the change in acetabular anteversion was approximately 0.4° for every 1° of rotation on the coronal plane of the pelvis and approximately 0.77° for every 1° of rotation on the sagittal plane of the pelvis, while the rotation of the pelvis on the horizontal plane had no effect on the measurement of acetabular anteversion^34^. Siebenrock et al.^24^ found that the pelvis between individuals in the natural prone position had a reclination of 12° and an inclination of 9° on the sagittal plane. This change makes the measurement of acetabular anteversion vary greatly among individuals. In this study, patient pelvic obliquity was less than 3°, which reduced the variation in acetabulum anteversion in the same individual and may be why there was no significant difference between the groups.

The results showed that the average age of the secondary ossification center was 8–14 years old, and the fusion of the secondary ossification center occurred earlier than Y-type cartilage closure^35–37^. The age of the secondary ossification center of the acetabulum edge overlaps with the age of Perthes disease. In the process of growth and development, Y-type cartilage only increases the width of the acetabulum but does not increase the depth of the acetabulum^38^, while the appearance of the secondary ossification of the acetabulum edge has a greater impact on the A and P. In 2014, Shapiro et al.^16^ found that Perthes disease caused acetabular morphological changes in an animal research model. Upasani et al.^17^ found that the coverage angle of the upper, posterior and lower quadrants of the acetabulum on the operative side was significantly reduced and that there was a reduction in the acetabular anteversion and tilt angles with the same animal model. This change is similar to the change in the acetabulum in children with Perthes disease. The author speculated that acetabular retroversion in Perthes disease may be caused by femoral head necrosis, which affects the ossification of the posterior wall of the acetabulum in adolescence. When the posterior wall coverage is reduced, acetabulum retroversion appears. He proposed two hypotheses on the causes of acetabular retroversion in children with Perthes disease: first, the interaction between the deformed femoral head and the acetabulum stimulated or hindered the growth of different areas of the acetabulum edge; and second, the osteogenesis of acetabular cartilage and Y-shaped cartilage was abnormal due to the inflammatory factors caused by femoral head necrosis in the joint. Although many studies have shown that Perthes disease can affect Y-shaped cartilage, articular cartilage and the labrum^3,12^, it is not clear whether Perthes disease can cause the development of the secondary ossification center of the acetabulum edge. In this study, there was no difference in the A, P and AWDI between the affected side and the contralateral side as well as between the disease group and the control group, but it is interesting to note that the acetabulum of the affected side in the case group was wider than that of the contralateral side; this result was also observed in previous studies that showed that the W increased in the early stage of Perthes disease^2,39,40^. The increase in W will lead to an increase in acetabulum anteversion, which suggests that we should to pay attention to the influence of W on acetabulum retroversion. However, there was no difference in the AWDI between the two sides of the case group, indicating that the acetabulum widening caused by Perthes disease has little effect on acetabulum anteversion.

We know that the onset age, gender and lateral classification of the children are important factors for determining the prognosis of Perthes disease. Ezoe et al.^5^ found that acetabular retroversion in Perthes disease is associated with poor prognosis. In addition, Larson et al.^21^ found that the Pris sign rate was 90% (9/10) in the early stage of Perthes' disease, but it is still unclear whether the acetabular retroversion is caused by congenital deformity and bone ischemia or caused by femoral head necrosis and acetabular retroversion. At present, in our experiment, no correlation was found between AAA, IWAA and AWDI and age, gender and Herring lateral pillar classification. This may be due to the large differences in the sample size between our data groups and the large individual differences among children, so we failed to find a significant correlation. We need more sample sizes and cohort studies to find correlations in the future.

The cause of acetabular retroversion in children with Perthes disease is not clear. However, in the study of acetabular anteversion in DDH patients, it was found that the acetabulum of the affected side rotated inward due to the interaction with the femoral head, causing acetabular anteversion^41^. Many researchers believe that acetabular retroversion in Perthes disease is due to the interaction between the necrotic femoral head and the acetabulum^6–8^. Some scholars have also found that there is a high proportion of acetabular retroversion in the early stage of Perthes disease, so it is speculated that acetabular retroversion may be a congenital malformation, and the femoroacetabular impact caused by acetabular retroversion may cause femoral head ischemia^21,39,40^. In a study by Yoshida et al.^6^, the crossover sign-positive group had acetabular retroversion in the early stage of the disease. He believed that acetabular retroversion was similar to acetabular dysplasia, which persisted during the development process. In the negative crossover sign group, the affected acetabulum gradually showed retroversion, which may have been caused by the inflammation of the hip joint, changes in hemodynamics, or mechanical factors involving the interaction between the acetabulum and the deformed femoral head. In the poor prognosis group, acetabular retroversion occurred on both sides of the hip over time, which may have been caused by factors such as lumbar kyphosis, the mechanical stimulation of the triangular cartilage and cytokines and genes produced by synovitis. Kawahara et al.^8^ found that 45.8% of patients with Perthes disease had bilateral hip retroversion, so he proposed that Perthes disease caused the developmental abnormality of the whole pelvis during the development process. Studies have shown that acetabulum anteversion, pelvic wall anteversion and the acetabulum wall index are not significantly related to patient age, gender, stage, lateral pillar grading and Catterall grading, so it is impossible to determine whether acetabular retroversion is caused by Perthes disease.

This study was similar to most case–control studies in regard to the existence of selection bias and measurement bias. At the same time, due to the loss of some image data in this retrospective study, three-dimensional (3D) reconstruction failed to reach the standardized pelvic position, and more accurate measurements were carried out^24,42^. Although the indicators in this study could also be affected by body position and posture^43^, the measurement indicators on the left and right sides of the individual are less affected by body position. Similar to other CT measurement indexes, this study was not able to study acetabulum marginal cartilage, but the study of the osteoacetabulum can be used as the basis of acetabulum development research. At the same time, the degree of acetabular retroversion measured in the supine position does not represent that in the standing position^44,45^.

In the future, the study of acetabular retroversion should include dynamic measurements in physiological states. Another, 2-D imaging may lead to the measurement eror, especially when considering very sensitive relations between the hemipelvis wall and acetabular orientation. We hope to obtain more complete data and a larger sample size to reduce measurement errors in future studies. Compared with other studies, the measurement method in this study reflected the relationship between the anteroposterior wall of the acetabulum and the anteversion and retroversion of the hemipelvis, and the measurement method was simple and intuitive. If this method is combined with 3D reconstruction and MRI, it could be very helpful for the study of acetabular retroversion.

## Conclusion

As an anatomic deformity, acetabulum retroversion has a high incidence in patients with Perthes disease. In this study, the anteversion of the pelvic wall and the AWDI predicted the anteversion of the acetabulum well and could distinguish the parts that caused retroversion. In this group, the acetabulum of the affected side was more retroverted than that of the opposite side. This result may have been caused by the distal retroversion of the hemipelvis. We have to be aware that using these measurements without 3-D techniques may lead to a considerable bias. The IWAA and AWDI can be used as new parameters in future studies of acetabulum retroversion.
